# Prognostic value of left atrial reservoir strain and global work efficiency in medically managed secondary mitral regurgitation: a mediation analysis

**DOI:** 10.3389/fcvm.2026.1924498

**Published:** 2026-09-03

**Authors:** Kangla Liao, Chunhua Mo, Li Liu, Bi Huang, Suxin Luo

**Affiliations:** 1 Department of Cardiovascular Medicine, Cardiovascular Research Center, The First Affiliated Hospital of Chongqing Medical University, Chongqing, China; 2 Cardiovascular Disease Laboratory of Chongqing Medical University, Chongqing, China; 3 Teaching and Research Section of Internal Medicine, The First Affiliated Hospital of Chongqing Medical University, Chongqing, China

**Keywords:** global work efficiency, left atrial reservoir strain, mediation analysis, prognosis, secondary mitral regurgitation

## Abstract

**Aims:**

Left atrial reservoir strain (LASr) and global work efficiency (GWE) have individually demonstrated prognostic value in secondary mitral regurgitation (SMR), but their interrelationship and combined utility remain unclear. We aimed to investigate the independent prognostic value of LASr and GWE, and to explore whether GWE plays a role in the LASr–mortality association in medically managed SMR patients.

**Methods and results:**

This retrospective study included 399 patients with SMR (median age 70.0 years, 66.4% male) who were medically managed without valve intervention. LASr and GWE were measured using speckle-tracking echocardiography. Cox regression and mediation analyses were performed. During a median follow-up of 35.0 months, 103 deaths (25.8%) occurred. Both LASr (adjusted HR 0.891 per 1% increase, 95% CI 0.850–0.933, *P* < 0.001) and GWE (adjusted HR 0.920 per 1% increase, 95% CI 0.891–0.951, *P* < 0.001) independently predicted all-cause mortality. Exploratory mediation analysis showed that GWE statistically accounted for a portion of the LASr–mortality association, with an indirect effect of −0.183 (95% CI −0.305 to −0.112, *P* < 0.001) and a mediation proportion of 16.3%.

**Conclusion:**

In medically managed SMR patients, both LASr and GWE independently predict all-cause mortality, and GWE statistically accounts for the relationship between LASr and mortality. These findings generate the hypothesis that left atrial dysfunction is statistically associated with impaired myocardial work efficiency, supporting the integrated assessment of both parameters for risk stratification across different SMR severity and phenotypes.

## Graphical Abstract

**Figure:**
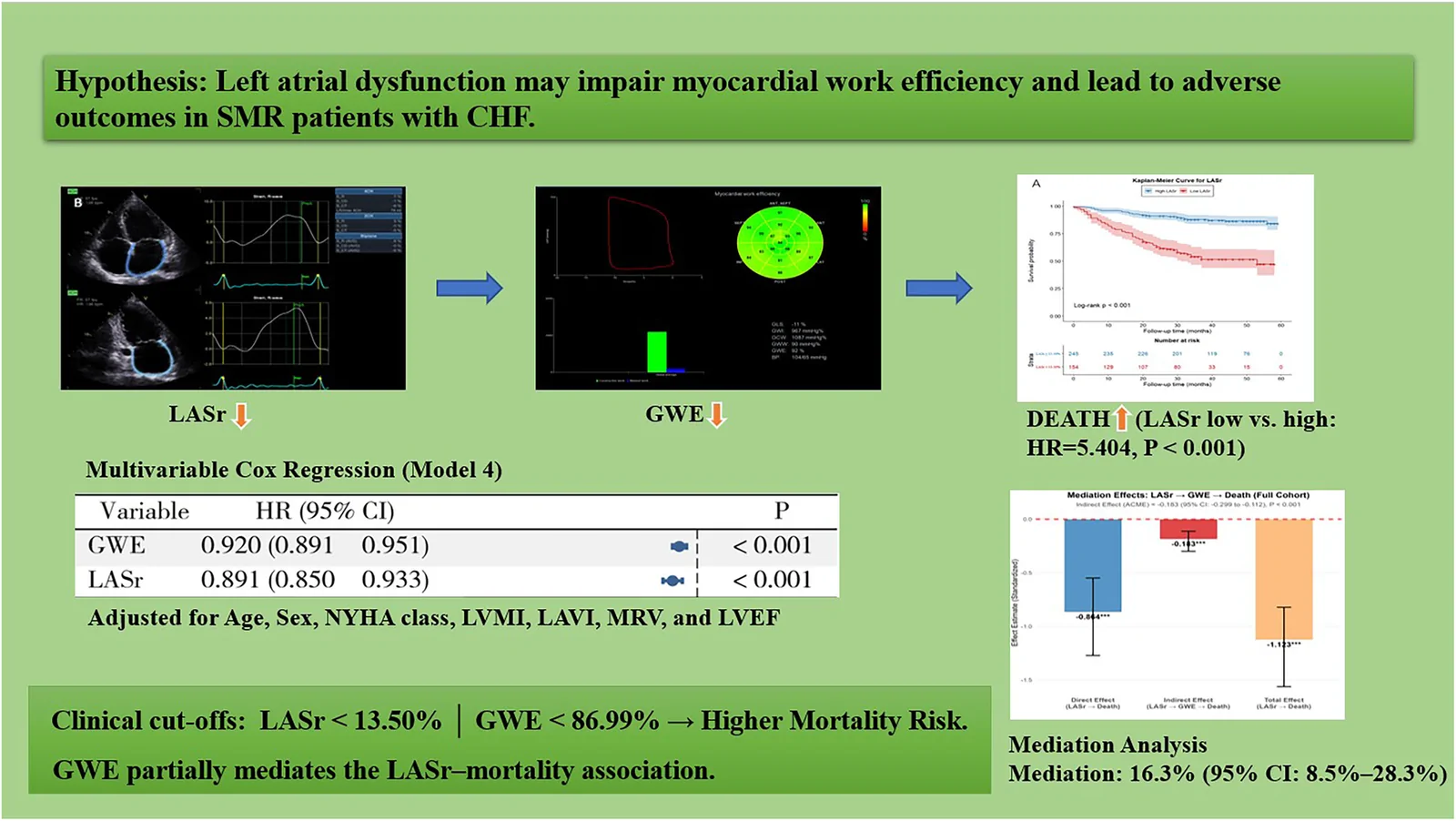

## Introduction

Secondary mitral regurgitation (SMR) is the most common and under-treated form of mitral regurgitation (1). It is highly prevalent in heart failure with reduced ejection fraction (HFrEF) and is strongly associated with increased mortality, heart failure rehospitalization, and poor quality of life (2, 3). Mechanistically, SMR arises from imbalance between leaflet tethering and closing forces, leading to malcoaptation, with left ventricular dysfunction and adverse remodeling as core drivers (4, 5). SMR encompasses distinct phenotypes: atrial SMR (aSMR) features marked left atrial and annular dilation with preserved left ventricular function (6, 7), whereas ventricular SMR (vSMR) is driven by left ventricular dilation, systolic dysfunction, and distorted geometry (8). Hemodynamically, SMR is classified as “proportional” (regurgitant volume consistent with ventricular dilation) or “disproportional” (excess regurgitation), the latter potentially warranting earlier intervention (1). Although the understanding of the pathophysiology of SMR has been continuously deepened, its clinical management still faces challenges. Particularly, for specific SMR phenotypes, there is currently no consensus on how to make a choice among different treatment strategies [including transcatheter edge-to-edge repair (TEER), annuloplasty or valve replacement surgery] (7, 9). This uncertainty underscores the critical need for improved risk stratification tools in SMR patients.

Assessment of left ventricular (LV) function in patients with secondary mitral regurgitation (SMR) remains challenging but is critical for risk stratification (10). Left ventricular ejection fraction (LVEF), a conventional metric, is limited by its load dependency, which may mask early myocardial dysfunction or overestimate contractile reserve under altered loading conditions (11, 12). Global work efficiency (GWE), a novel index derived from pressure-strain loop analysis, integrates myocardial deformation with afterload, thereby offering a more comprehensive assessment of LV performance independent of loading status (10, 12). In SMR patients, reduced GWE is independently associated with excess mortality (10), and impaired global work efficiency predicts worse outcomes, supporting its prognostic value (13). Furthermore, GWE dynamically tracks changes following valve intervention in a study of AR patients, suggesting its sensitivity to load-dependent modifications and its potential to overcome LVEF limitations (14). Thus, GWE may serve as a more precise and sensitive prognostic marker in SMR. Global longitudinal strain (GLS), a conventional measure of LV deformation, has been widely used for risk stratification in heart failure, but it remains load-dependent and does not account for myocardial work efficiency (15). GWE, which integrates strain with estimated LV pressure, may overcome some of these limitations (15).

Left atrial reservoir strain (LASr) has emerged as a sensitive marker of left atrial dysfunction and elevated left ventricular filling pressure, providing incremental diagnostic and prognostic value over conventional parameters (16, 17). In patients with significant secondary mitral regurgitation, impaired LASr is independently associated with excess all-cause mortality (18). Notably, LASr has been shown to offer incremental prognostic value beyond left atrial volume and left ventricular global longitudinal strain, particularly in patients with atrial fibrillation (18, 19). However, whether GWE mediates the relationship between LASr and mortality in SMR remains unknown.

Although both LASr and GWE have individually demonstrated prognostic value in heart failure and valvular heart disease, their combined utility and the potential mediating role of GWE in the LASr-mortality relationship in SMR have not been investigated. We hypothesized that GWE may partially statistically account for the association between LASr and mortality, consistent with the concept that left atrial dysfunction is related to impaired ventricular filling and myocardial work efficiency, which may in turn be associated with adverse outcomes. Therefore, this study aimed to: (1) quantify the independent prognostic value of LASr and GWE in patients with SMR, and (2) explore, within a statistical mediation framework, whether GWE statistically accounts for part of the association between LASr and all-cause mortality.

## Materials and methods

### Study population

This retrospective, single-center observational study was conducted at the Department of Cardiology, the First Affiliated Hospital of Chongqing Medical University. Between April 2021 and October 2024, a total of 500 consecutive patients with chronic heart failure (CHF) and secondary mitral regurgitation (SMR) were screened. Inclusion criteria were: (1) age ≥ 18 years; (2) confirmed diagnosis of CHF; and (3) SMR of at least mild severity, regardless of left ventricular ejection fraction (LVEF) status; (4) absence of organic valvular heart disease (including valve stenosis, significant aortic valve regurgitation or tricuspid valve regurgitation) and no right ventricular cardiomyopathy or significant pulmonary hypertension (> 70mmHg); and (5) no prior cardiac resynchronization therapy (CRT) or implantable cardioverter-defibrillator (ICD) implantation. Exclusion criteria applied during follow-up included: acute myocardial infarction (*n* = 5), hypertrophic cardiomyopathy (*n* = 11), poor echocardiographic image quality or heart rate variability precluding reliable speckle-tracking strain analysis (*n* = 37), missing blood pressure measurements at examination (*n* = 16), valve surgery during follow-up (*n* = 4), and loss to follow-up (*n* = 28). After applying these criteria, 399 patients were included in the final analysis. During a median follow-up of 35.0 months, 103 deaths (25.8%) occurred.

The study protocol was approved by the Institutional Ethics Committee of the First Affiliated Hospital of Chongqing Medical University (Approval No. 2021-132, dated March 3, 2021), and all participants provided written informed consent.

### Clinical data collection

Demographic characteristics, comorbidities, laboratory parameters (including blood glucose, lipid profile, and N-terminal pro-B-type natriuretic peptide [NT-proBNP]), and discharge medication regimens were extracted from electronic medical records. Medication regimens were recorded at discharge after in-hospital optimization according to contemporary heart failure guidelines.

### Echocardiographic data acquisition and analysis

Transthoracic echocardiography was performed by experienced sonographers using a GE Vivid E95 ultrasound system (GE Vingmed Ultrasound, Horten, Norway) with continuous electrocardiographic monitoring. Image acquisition employed an S5 transducer (1.5–4.6 MHz), and frame rates were maintained between 50 and 70 frames per second. Participants were positioned in the left lateral decubitus position throughout the examination. Standard two-dimensional cineloops from the apical four-chamber, two-chamber, and long-axis views were acquired following contemporary American Society of Echocardiography and European Association of Cardiovascular Imaging guidelines (20). For each view, three consecutive cardiac cycles were captured in patients with sinus rhythm, while five consecutive cycles were acquired in those with atrial fibrillation, with all recordings obtained during stable heart rhythm. Accepted images met the following quality criteria: clearly delineated endocardial borders and less than 5% heart rate variability.

Offline speckle-tracking analysis was performed using EchoPAC software (GE Vingmed Ultrasound) by two independent investigators blinded to clinical outcomes. LVEF was measured using the biplane Simpson method. Left ventricular mass index (LVMI) and left atrial volume index (LAVI) were indexed to body surface area. Mitral regurgitation volume (MRV) and effective regurgitant orifice area (EROA) were assessed via the proximal isovelocity surface area (PISA) method. Vena contracta width (VCW) was measured as the narrowest portion of the MR jet at the level of the mitral leaflets in the parasternal long-axis view. PISA radius was measured at the onset of systole. E/e' was calculated as the ratio of early diastolic transmitral flow velocity (E) to early diastolic mitral annular velocity (e'). GLS was measured using speckle-tracking echocardiography from the apical four-chamber, two-chamber, and three-chamber views.

**Figure 1:**
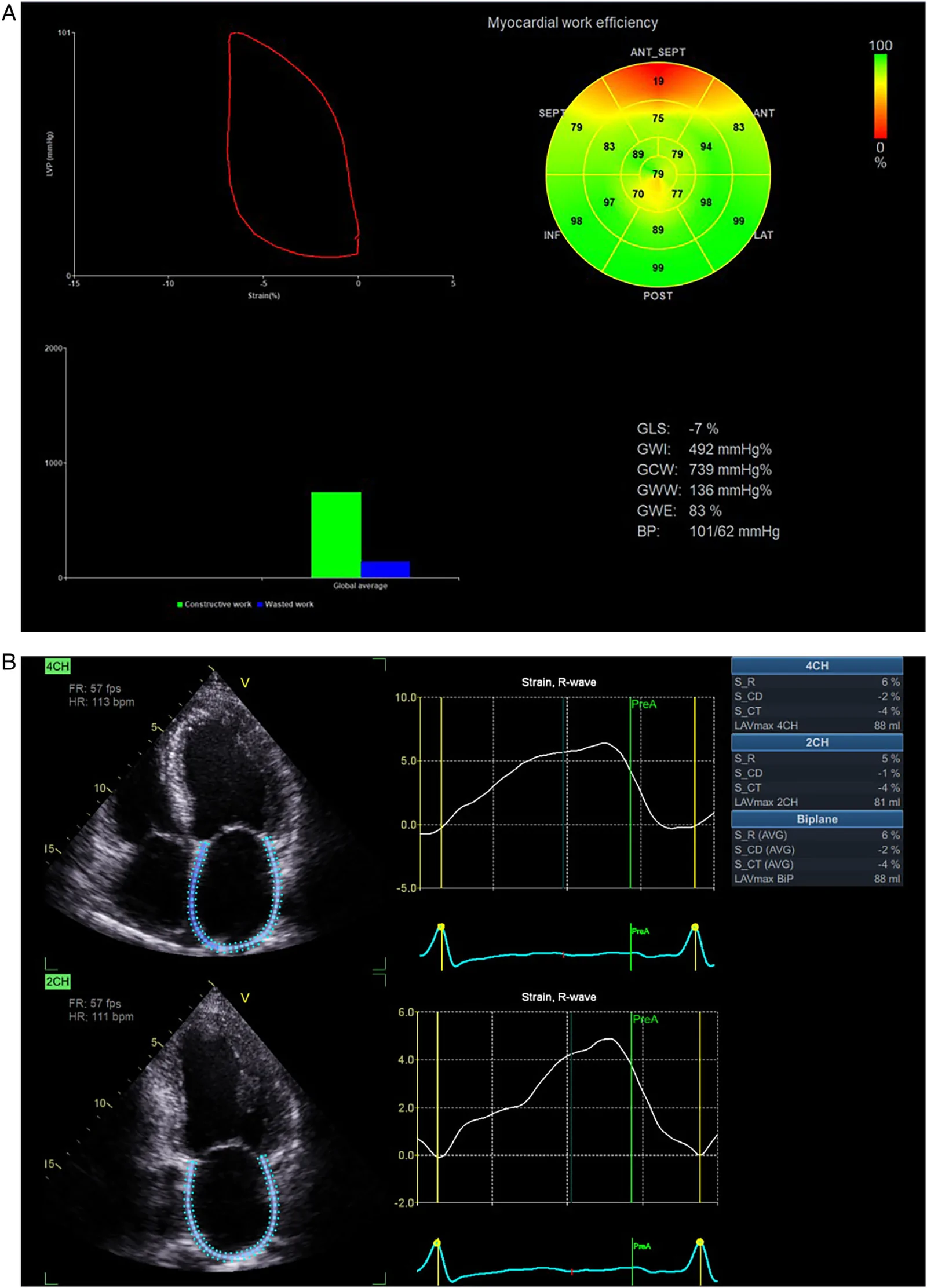
Representative speckle-tracking echocardiography images for GWE and LASr. **(A)** Bull's eye plot of global work efficiency (GWE) showing segmental distribution of myocardial work efficiency. **(B)** Left atrial reservoir strain (LASr) analysis from the apical four-chamber and two-chamber views. The strain curve depicts phasic changes in left atrial deformation; the peak during ventricular systole (LASr) reflects left atrial reservoir function.

Myocardial work parameters, including global work index (GWI), global constructive work (GCW), global wasted work (GWW), and global work efficiency (GWE), were derived from pressure-strain loop analysis by combining speckle-tracking-derived strain with estimated left ventricular pressure using a non-invasive brachial cuff pressure waveform. LASr was measured from the left atrium using the apical four-chamber and two-chamber views. The region of interest was manually traced along the left atrial endocardial border, excluding the pulmonary veins and left atrial appendage. LASr was defined as the peak positive strain during ventricular systole, reflecting left atrial reservoir function (Figure 1).

Based on clinical history, coronary angiography, and echocardiographic findings, the etiology of secondary mitral regurgitation (SMR) was classified as ischemic (prior myocardial infarction or significant coronary artery disease) or non-ischemic (e.g., dilated cardiomyopathy, hypertensive heart disease, or atrial fibrillation) (8). In addition, patients were categorized according to SMR phenotype using previously published criteria (21, 22): atrial SMR (aSMR) was defined as left ventricular ejection fraction (LVEF) ≥ 50% with left atrial enlargement (LAVI >34 mL/m^2^), and absence of significant LV remodeling; ventricular SMR (vSMR) was defined as LVEF <50% and/or left ventricular dilatation (LVEDD >55 mm in men and >50 mm in women); and a mixed phenotype was assigned when both criteria were met simultaneously.

### Study endpoint

Survival status was ascertained through electronic medical records and supplemented by telephone interviews when annual outpatient follow-up data were unavailable. The final follow-up assessment was completed April 2026. The primary endpoint of the study was all-cause mortality.

### Statistical analysis

Continuous variables are presented as medians with interquartile ranges (IQR) and were compared between groups using the Mann–Whitney U test. Categorical variables are expressed as frequencies with percentages and were analyzed using the chi-square test or Fisher's exact test, as appropriate.

Receiver operating characteristic (ROC) curve analysis was performed to evaluate the discriminatory ability of LASr, GWE, and other echocardiographic parameters for all-cause mortality. The optimal cutoff values were determined using the Youden index (maximizing sensitivity+specificity−1). Area under the curve (AUC) values were compared using DeLong's method for paired ROC curves.

Kaplan–Meier survival curves were constructed to compare survival probabilities between groups stratified by the optimal cutoff values of LASr and GWE, with differences assessed using the log-rank test. Univariate Cox regression analyses were performed within each stratum to estimate hazard ratios (HRs). Prior to analysis, NT-proBNP was natural log-transformed due to its skewed distribution.

Cox proportional hazards regression models were constructed to identify independent predictors of all-cause mortality. Variables included in the multivariable models were selected based on clinical relevance and prior evidence of association with prognosis in heart failure. Five hierarchical models were fitted:

Model 1 (Basic): adjusted for sex, age, NYHA class (III–IV vs. I–II), LVMI, LAVI, and MRV.
Model 2 (+LVEF): Model 1 plus LVEF.
Model 3 (+GWE): Model 2 plus GWE.
Model 4 (+LASr): Model 3 plus LASr.
Model 5 (+Interaction): Model 4 plus the GWE x LASr interaction term.

Model discrimination was assessed using Harrell's C-index. Model fit was compared using Akaike's information criterion (AIC) and likelihood ratio tests for nested models. Differences in C-index between models were evaluated using DeLong's method for paired samples.

To further address the concern of residual confounding, an additional sensitivity model was constructed incorporating NT-proBNP, eGFR, SBP, ischemic etiology, TAPSE, TR velocity, and Guideline-Directed Medical Therapy (GDMT) score, in addition to the core covariates (LVEF, atrial fibrillation [AF], LAVI) and the primary predictors (LASr, GWE). Due to the limited number of events and the exploratory nature of this analysis, the results are presented. Given the limited events, this model was prespecified as exploratory.

Integrated discrimination improvement (IDI) was calculated to evaluate the incremental prognostic value of adding LASr, GWE, or both to the basic model. IDI estimates and 95% confidence intervals were derived from 1,000 bootstrap resamples.

Prespecified subgroup analyses were performed for sex, age (≤70 vs. > 70 years, using the median as cutoff), LVEF (≥50% vs. < 50%), NYHA class (I/II vs. III/IV), LVMI (≤144.55 vs. > 144.55 g/m^2^, using the median as cutoff), LAVI using two cutoffs: ≤34 vs. > 34 mL/m^2^(defined as LA enlargement) and ≤48 vs. > 48 mL/m^2^ (defined as severe LA enlargement, used for the EROA subgroup analysis and multivariable adjustment), and AF status (no vs. yes). For each subgroup, univariate Cox regression was used to estimate the association of LASr and GWE with mortality. Interaction terms between the predictor (LASr or GWE) and each subgroup variable were introduced into separate Cox models to test for effect modification. *To further assess the robustness of MR severity as a quantitative parameter, subgroup analyses were also performed for EROA across the same stratification variables (sex, age, LVEF, NYHA, AF, LVMI, and LAVI). EROA was analyzed as a continuous variable, and interaction terms between EROA and each subgroup variable were tested using separate Cox models.*

A simplified GDMT score was calculated based on the use of ACEi/ARB/ARNI (1 point) and beta-blockers (1 point), resulting in a score ranging from 0 to 2. This scoring approach is consistent with established GDMT scoring frameworks that identify RAS inhibitors and beta-blockers as the core foundational components of heart failure pharmacotherapy (23). MRAs and SGLT2 inhibitors were not included due to the low prescription rates in this cohort and the fact that SGLT2 inhibitors had not been widely adopted during the study period.

Prior to mediation analysis, partial regression analysis adjusted for covariates was used to confirm the independent linear association between LASr and GWE. An exploratory mediation analysis was then performed to examine whether GWE mediates the association between LASr and all-cause mortality. The mediation model consisted of: (1) a mediator model regressing GWE on LASr, and (2) an outcome model regressing survival on both LASr and GWE. All models were adjusted for age, sex, NYHA class, LVMI, LAVI, MRV, and LVEF. The average causal mediation effect (ACME), average direct effect (ADE), total effect, and proportion mediated were estimated using bootstrap resampling with 1,000 replicates. For residual-based validation, the GWE residual was standardized (mean 0, SD 1) prior to Cox regression, and the HR represents the change in mortality risk per 1-SD increase in the residual component of GWE independent of LASr. Importantly, given the cross-sectional nature of LASr and GWE measurements, this mediation analysis should be interpreted as exploratory and hypothesis-generating rather than as proof of causation. The validity of the mediation model relies on assumptions including no unmeasured confounding of the exposure–mediator and mediator–outcome relationships, which cannot be fully verified in observational data. We therefore emphasize that the mediation findings are presented as a statistical framework to generate hypotheses for future investigation.

To address the possibility that inclusion of mild MR might dilute the clinical relevance of our findings, we performed a sensitivity analysis by repeating the main prognostic analyses (ROC, Kaplan–Meier, univariate and multivariable Cox regression, and mediation analysis) after excluding patients with mild MR (MRV < 30 mL), restricting the cohort to those with moderate-to-severe SMR.

All statistical tests were two-sided, and a *P* value < 0.05 was considered statistically significant. Analyses were conducted using Prism 10 and R version 4.5.2 (R Foundation for Statistical Computing, Vienna, Austria) with the following packages: survival, pROC, ggplot2, dplyr, mediation, and Hmisc, and forestplot.

## Results

### Baseline characteristics

A total of 399 patients with SMR were included in the final analysis. The median age was 70.0 years (interquartile range [IQR]: 60.0–77.0), and 265 patients (66.4%) were male. During a median follow-up of 35.0 months (IQR: 16.0–44.0), 103 deaths (25.8%) occurred.

Table 1 compares baseline clinical and echocardiographic characteristics between survivors (*n* = 295) and non-survivors (*n* = 103). Non-survivors had a higher proportion of NYHA class III–IV (69.9% vs. 48.0%, *P* = 0.001) and more frequent diuretic use (67.0% vs. 41.6%, *P* < 0.001), while CCB use was less common in non-survivors (37.9% vs. 53.0%, *P* = 0.008). Additionally, non-survivors presented with lower systolic blood pressure (117 vs. 121 mmHg, *P* = 0.034). No significant differences were observed in age, sex, comorbidities, or other medications between the two groups (all *P* > 0.05). Echocardiographic evaluation revealed that non-survivors had lower LVEF (32.0% vs. 41.0%, *P* < 0.001), larger LVEDD (62.0 vs. 56.5 mm, *P* < 0.001) and LVESD (51.0 vs. 45.0 mm, *P* < 0.001), higher LVMI (160.8 vs. 139.9 g/m^2^, *P* < 0.001), higher LAVI (54.6 vs. 47.5 mL/m^2^, *P* = 0.001), higher E/e' (17.0 vs. 15.3, *P* = 0.007), and higher MRV (34.0 vs. 27.0 mL, *P* < 0.001) as well as greater EROA (30.4 vs. 23.3 mm^2^, *P* < 0.001), VCW (5.4 vs. 4.4 mm, *P* < 0.001), and PISA radius (0.9 vs. 0.8 cm, *P* < 0.001) compared with survivors. Regarding myocardial work parameters, non-survivors had lower GWE (83.8% vs. 89.4%, *P* < 0.001), lower GWI (687.0 vs. 948.5 mmHg%, *P* < 0.001), lower GCW (1,047.0 vs. 1,295.5 mmHg%, *P* < 0.001), and higher GWW (213.0 vs. 144.0 mmHg%, *P* < 0.001), GLS was reduced in non-survivors (8.3% vs. 11.9%, *P* < 0.001). LASr was lower in non-survivors (10.0% vs. 17.0%, *P* < 0.001). Regarding SMR etiology and phenotype, non-survivors had a higher prevalence of ischemic SMR (48.5% vs. 32.1%, *P* = 0.003) and a significantly higher proportion of dual functional MR (78.6% vs. 57.8%, *P* < 0.001), whereas AFMR was less common in non-survivors (6.8% vs. 21.6%, *P* < 0.001).

**Table 1:** Baseline clinical and echocardiographic characteristics of the study population stratified by survival Status.

Characteristics	Survivors	Non-survivors	*P* value
N	295	103	
Clinical characteristics			
Time, m	40.00 (33.00, 51.00)	16.00 (7.00, 24.00)	<0.001
male, *n* (%)	199 (67.20)	66 (64.10)	0.560
age, y	70.00 (60.00, 77.00)	70.00 (62.00, 77.00)	0.943
BSA, m^2^	1.66 (1.54,1.75)	1.64 (1.49,1.77)	0.223
SBP, mmHg	121.00 (108.00, 131.00)	117.00 (101.00, 129.00)	0.034
DBP, mmHg	75.00 (67.00, 83.00)	72.00 (66.00, 81.00)	0.179
Heart, bpm	76.00 (64.00, 87.00)	76.00 (65.00, 89.00)	0.600
Comorbidities, *n* (%)			
Hypertension	166 (56.10)	49 (47.60)	0.136
CHD	174 (58.80)	50 (48.50)	0.071
DM	97 (32.80)	28 (27.20)	0.292
AF	99 (33.40)	29 (28.20)	0.322
NYHA, *n* (%)			0.001
I	75 (25.3%)	11 (10.7%)	
II	79 (26.7%)	20 (19.4%)	
III	110 (37.2%)	53 (51.5%)	
IV	32 (10.8%)	19 (18.4%)	
Laboratory data			
Cr, mg/dL	84.00 (69.00, 118.00)	83.00 (66.00, 123.00)	0.911
eGFR, mL/min/1.73m^2^	75.30 (50.60, 90.88)	73.80 (44.50, 88.40)	0.416
HB, g/L	133.00 (120.00, 146.00)	134.00 (118.00, 147.00)	0.929
NT-proBNP, pg/mL	2,870.00 (1,040.00, 7,860.00)	3,280.00 (1,200.00, 6,760.00)	0.786
TC, mmol/L	3.69 (3.10, 4.46)	3.52 (3.05, 4.25)	0.205
TG, mmol/L	1.11 (0.84, 1.52)	1.18 (0.86, 1.58)	0.516
HDLC, mmol/L	1.06 (0.87, 1.31)	0.98 (0.83, 1.22)	0.068
LDLC, mmol/L	2.22 (1.69, 2.83)	2.09 (1.65, 2.78)	0.295
Medications, *n* (%)			
ARNI/ACEI/ARB	230 (77.70)	80 (77.70)	0.995
CCB	157 (53.00)	39 (37.90)	0.008
Beta-blocker	232 (78.40)	74 (84.10)	0.177
Antiplatelet	183 (61.80)	56 (54.40)	0.184
Statins	191 (64.50)	57 (55.30)	0.098
Diuretic	123 (41.60)	69 (67.00)	<0.001
Echocardiographic characteristics			
LVEF, %	41.00 (31.00, 57.00)	32.00 (26.00, 42.00)	<0.001
LVEDD, mm	56.50 (51.00, 64.38)	62.00 (54.00, 66.90)	<0.001
LVESD, mm	45.00 (36.00, 54.75)	51.00 (42.60, 57.90)	<0.001
LVMI, g/m^2^	139.91 (113.43, 166.13)	160.84 (134.71, 194.17)	<0.001
LAVI, mL/m^2^	47.49 (35.74, 59.25)	54.61 (41.67, 73.38)	0.001
E/e'	15.29 (11.44, 20.64)	17.00 (13.05, 23.33)	0.007
TR, cm/s	2.65 (2.31, 3.09)	2.75 (2.49, 3.18)	0.172
TAPSE, mm	16.00 (13.90,17.50)	15.95 (12.85, 17.43)	0.235
MRV, mL	27.00 (16.00, 37.00)	34.00 (26.00, 50.00)	<0.001
EROA, mm^2^	23.30 (12.35, 31.75)	30.40 (22.20, 38.40)	<0.001
VCW, mm	4.40 (3.60, 6.20)	5.40 (4.30, 7.50)	<0.001
PISA radius, cm	0.80 (0.60, 0.90)	0.90 (0.70, 1.00)	<0.001
Myocardial work parameters			
GLS, %	11.85 (7.63, 16.10)	8.30 (5.70, 10.70)	<0.001
GWI, mmHg%	948.50 (605.50, 1,501.00)	687.00 (469.00, 969.00)	<0.001
GCW, mmHg%	1,295.50 (890.75, 1,872.00)	1,047.00 (793.00, 1,439.00)	<0.001
GWW, mmHg%	144.00 (102.25, 199.50)	213.00 (153.00, 275.00)	<0.001
GWE, %	89.41 (86.32, 92.12)	83.84 (78.55, 86.98)	<0.001
LASr, %	17.00 (13.00, 23.00)	10.00 (7.00, 15.00)	<0.001
SMR etiology and phenotype			
Etiology, *n* (%)			
Ischemic SMR	95 (32.1%)	50 (48.5%)	0.003
Non-ischemic SMR	201 (67.9%)	53 (51.5%)	
Phenotype, *n* (%)			
AFMR	64 (21.6%)	7 (6.8%)	<0.001
VFMR	61 (20.6%)	15 (14.6%)	
Dual FMR	171 (57.8%)	81 (78.6%)	

BSA, body surface area; SBP, systolic blood pressure; DBP, diastolic blood pressure; CHD, coronary heart disease; DM, diabetes mellitus; AF, atrial fibrillation; NT-proBNP, N-terminal pro-B type natriuretic peptide; NYHA, New York Heart Association functional classification; HB, hemoglobin; Cr, creatinine; TC, total cholesterol; TG, triglycerides; HDLC, high-density lipoprotein cholesterol; LDLC, low-density lipoprotein cholesterol; ARNI, angiotensin receptor & neprilysin inhibitor; ACEI, angiotensin converting enzyme inhibitor; ARB, angiotensin receptor blocker; CCB, calcium channel blocker; LVEF, left ventricular ejection fraction; LVEDD, left ventricular end-diastolic diameter; LVESD, left ventricular end-systolic diameter; LVMI, left ventricular mass index; LAVI, left atrial volume index; E/e’, ratio of early diastolic mitral inflow velocity to early diastolic mitral annular velocity; MRV, mitral regurgitation volume; EROA, effective regurgitant orifice area; VCW, vena contracta width; LASr, left atrial reservoir strain; GLS, global longitudinal strain; GWI, global work index; GCW, global constructive work; GWW, global wasted work; GWE, global work efficiency; AFMR, atrial functional mitral regurgitation; VFMR, ventricular functional mitral regurgitation; Dual FMR, dual functional mitral regurgitation.

### Correlation between LASr and GWE

**Figure 2:**
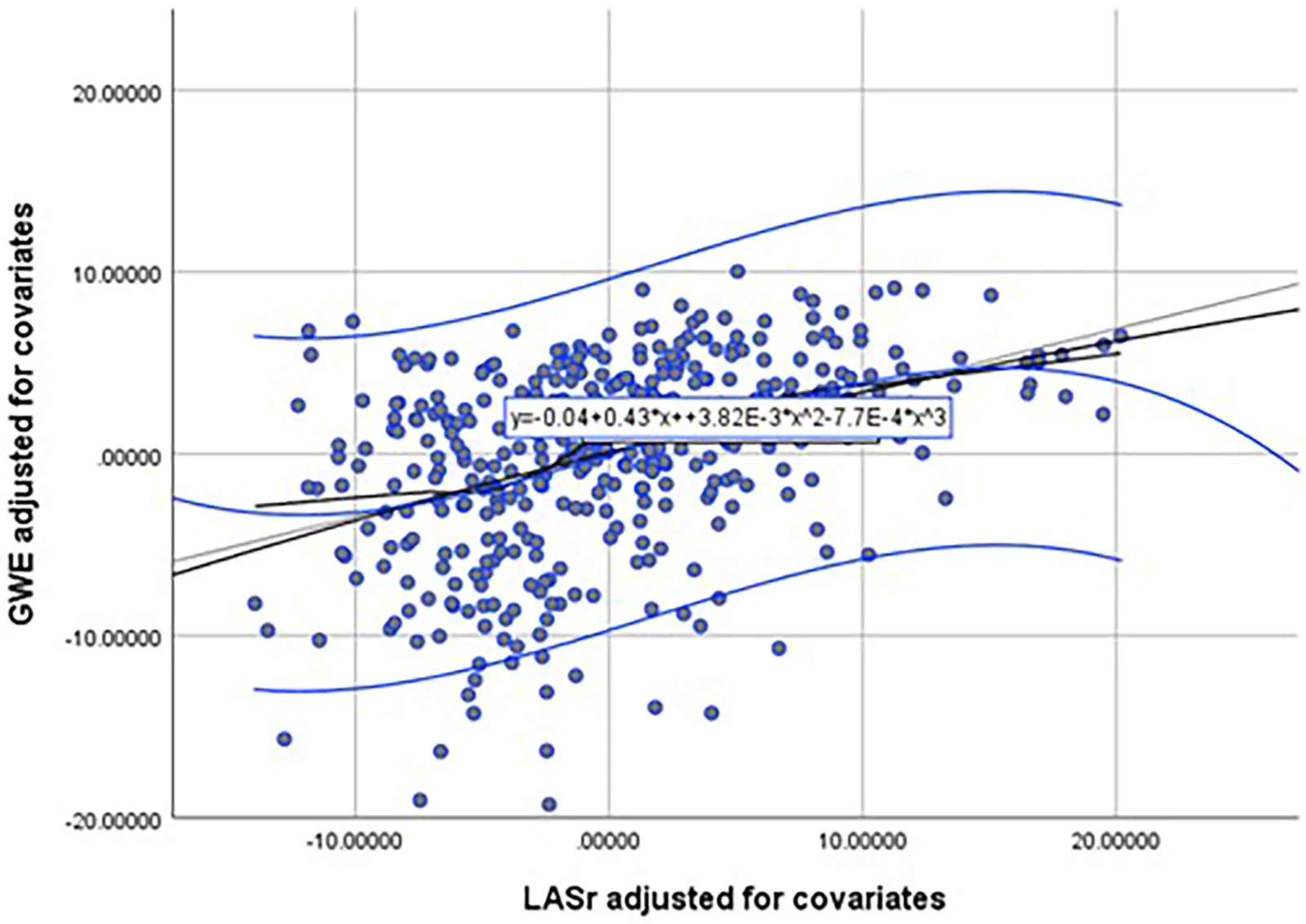
Partial regression plot of left atrial reservoir strain (LASr) versus global work efficiency (GWE). The plot illustrates the independent association between LASr and GWE after adjustment for LVEF, age, sex, systolic blood pressure, heart rate, and NYHA functional class. The *X*-axis and *Y*-axis represent the residuals of LASr and GWE, respectively, after accounting for the aforementioned covariates. The solid line represents the linear regression fit (y = –0.04 + 0.43x), with the gray shaded area indicating the 95% confidence interval. The R^2^ values for linear, quadratic, and cubic models were 0.165, 0.165, and 0.171, respectively, confirming a linear relationship between LASr and GWE, with no additional goodness-of-fit provided by curvilinear models.

Figure 2 shows the partial regression plot of LASr versus GWE after adjustment for LVEF, age, sex, systolic blood pressure, heart rate, and NYHA functional class. LASr was independently associated with GWE (*β* = 0.43, 95% CI: 0.35–0.51, *P* < 0.001). The R^2^ values for linear, quadratic, and cubic models were 0.165, 0.165, and 0.171, respectively, indicating a linear relationship without additional fit from curvilinear models.

### Prognostic value of LASr and GWE

#### Kaplan–Meier survival analysis

**Figure 3:**
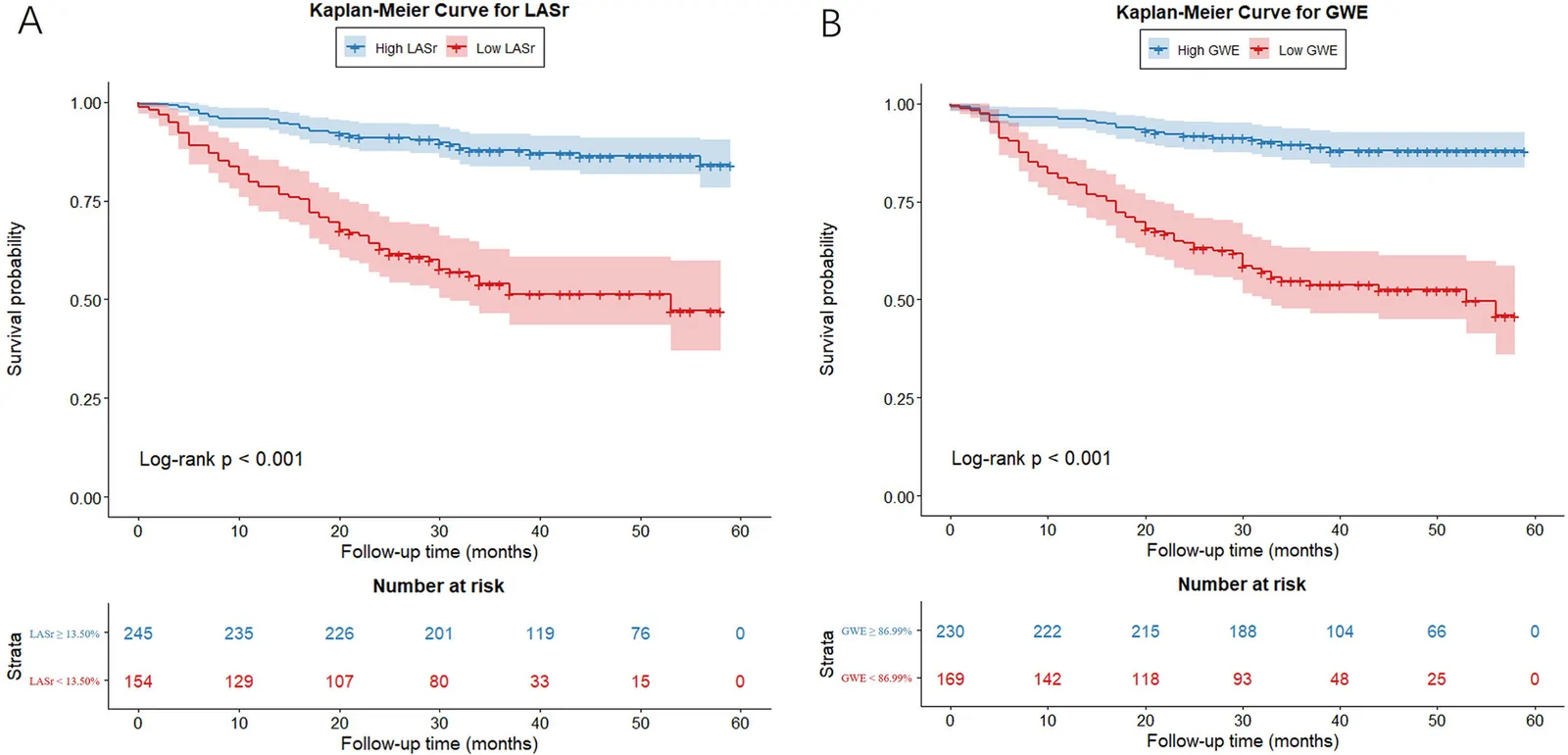
Kaplan–meier survival curves for all-cause mortality according to LASr and GWE levels. **(A)** Survival curves for patients stratified by LASr levels (high: ≥13.50%; low: <13.50%). **(B)** Survival curves for patients stratified by GWE levels (high: ≥86.99%; low: <86.99%). Log-rank tests demonstrated significantly lower survival probabilities in the low LASr and low GWE groups compared with the high LASr and high GWE groups, respectively (both *P* < 0.001). Hazard ratios (HRs) were calculated using univariate Cox regression analysis. LASr low vs. high: HR = 5.404 (95% CI: 3.544–8.242), *P* < 0.001. GWE low vs. high: HR = 5.679 (95% CI: 3.785–8.520), *P* < 0.001. Number at risk at each time point is shown below the curves.

Figure 3 presents the Kaplan–Meier survival curves for all-cause mortality stratified by LASr and GWE levels. Using the exploratory optimal cutoff values derived from ROC analysis (LASr: 13.50%; GWE: 86.99%), patients were divided into high and low groups. Patients with low LASr (<13.50%) had significantly worse survival than those with high LASr (≥13.50%), with a 5.40-fold increased risk of all-cause mortality (HR = 5.404, 95% CI: 3.544–8.242, log-rank *P* < 0.001). The median survival time was 44.0 months (95% CI: 37.0–51.0) in the low LASr group, whereas the median survival was not reached in the high LASr group (Figure 3A). Similarly, patients with low GWE (<86.99%) had significantly worse survival than those with high GWE (≥86.99%), with a 5.68-fold increased risk of all-cause mortality (HR = 5.679, 95% CI: 3.785–8.520, log-rank *P* < 0.001). The median survival time was 40.0 months (95% CI: 33.0–47.0) in the low GWE group, whereas the median survival was not reached in the high GWE group (Figure 3B).

### ROC analysis

Supplementary Figure S1 and Supplementary Table S1 show the ROC analysis for predicting all-cause mortality. GWE showed an AUC of 0.788 (95% CI: 0.739–0.838), and LASr showed an AUC of 0.779 (95% CI: 0.728–0.830). The DeLong test indicated no significant difference between the two AUCs (*P* = 0.759). The optimal cutoff values were 13.50% for LASr (sensitivity: 0.689, specificity: 0.720) and 86.99% for GWE (sensitivity: 0.757, specificity: 0.693). Other parameters, including GWW (AUC=0.704), GLS (AUC = 0.688), LVEF (AUC = 0.676), MRV (AUC = 0.675), GWI (AUC = 0.643), and GCW (AUC = 0.616), showed lower AUCs.

### Multivariable Cox regression analysis

Table 2 summarizes five multivariable Cox regression models. In Model 1, MRV was associated with increased mortality risk (HR = 1.023, 95% CI: 1.010–1.035, *P* < 0.001). In Model 2, LVEF was associated with reduced risk (HR = 0.955, 95% CI: 0.934–0.977, *P* < 0.001). In Model 3, GWE was independently associated with reduced risk (HR = 0.894, 95% CI: 0.867–0.923, *P* < 0.001); LVEF and MRV remained significant. In Model 4, LASr was independently associated with reduced risk (HR = 0.891, 95% CI: 0.850–0.933, *P* < 0.001); GWE, LVEF, and MRV remained significant. In Model 5, the GWE × LASr interaction term was not significant (HR = 1.001, 95% CI: 0.995–1.007, *P* = 0.764).

**Table 2:** Multivariable Cox regression models for predicting all-cause mortality.

Variable	Model 1 (Basic)	Model 2 (+LVEF)	Model 3 (+GWE)	Model 4 (+LASr)	Model 5 (+Interaction)
Sex	0.816 (0.540–1.233)	0.791 (0.523–1.198)	0.989 (0.649–1.507)	1.174 (0.765–1.801)	1.178 (0.767–1.809)
Age	1.003 (0.988–1.019)	1.010 (0.994–1.027)	1.009 (0.992–1.027)	1.008 (0.991–1.025)	1.008 (0.991–1.025)
NYHA III–IV	1.549 (0.981–2.445)	1.181 (0.747–1.867)	1.313 (0.839–2.055)	1.123 (0.716–1.761)	1.118 (0.712–1.756)
LVMI	1.007 (1.002–1.011)**	1.001 (0.996–1.007)	0.999 (0.994–1.004)	0.999 (0.993–1.004)	0.999 (0.993–1.004)
LAVI	1.001 (0.998–1.004)	1.002 (1.000–1.005)	1.002 (0.999–1.005)	1.000 (0.996–1.004)	1.000 (0.997–1.004)
MRV	1.023 (1.010–1.035)***	1.022 (1.009–1.035)**	1.026 (1.012–1.039)***	1.015 (1.001–1.030)*	1.015 (1.001–1.030)*
LVEF	–	0.955 (0.934–0.977)***	0.973 (0.951–0.997)*	0.975 (0.953–0.998)*	0.975 (0.952–0.997)*
GWE	–	–	0.894 (0.867–0.923)***	0.920 (0.891–0.951)***	0.924 (0.881–0.970)**
LASr	–	–	–	0.891 (0.850–0.933)***	0.893 (0.849–0.938)***
GWE × LASr	–	–	–	–	1.001 (0.995–1.007)

Data are presented as hazard ratio (HR) with 95% confidence interval (CI). Model 1: adjusted for sex, age, NYHA (III–IV vs. I–II), LVMI, LAVI, and MRV. Model 2: Model 1 + LVEF. Model 3: Model 2 + GWE. Model 4: Model 3 + LASr. Model 5: Model 4 + GWE × LASr interaction term. *P < 0.05, **P < 0.01, ***P < 0.001.

NYHA, New York Heart Association functional classification; LVMI, left ventricular mass index; LAVI, left atrial volume index; MRV, mitral regurgitation volume; LVEF, left ventricular ejection fraction; GWE, global work efficiency; LASr, left atrial reservoir strain; HR, hazard ratio; CI, confidence interval.

Supplementary Table S2 compares model fit across the five nested models. Model 4 (+LASr) demonstrated the lowest AIC (1,068.09) and the highest C-index (0.7988), indicating the best overall model fit and discriminative ability. Adding the interaction term (Model 5) did not further improve model performance (Δ*χ*^2^ = 0.06, Δdf = 1, *P* = 0.764), supporting the use of the parsimonious Model 4 as the primary model. In addition, IDI analysis (Supplementary Table S3) showed that GWE significantly improved risk reclassification over the basic model (IDI = 0.061, 95% CI: 0.024–0.098, *P* < 0.001), whereas adding LASr to the GWE model did not provide further improvement (IDI=−0.027, 95% CI: −0.048 to −0.006, *P* = 0.016), consistent with the moderate correlation between LASr and GWE (*r* = 0.595).

To further assess the robustness of our findings against residual confounding, we constructed an extended multivariable model incorporating NT-proBNP, eGFR, SBP, ischemic etiology, AF, TAPSE, TR velocity, GDMT score, and MRV, in addition to LASr and GWE (Supplementary Table S4). In this model (*n* = 364, events=94, EPV=8.55), both LASr < 13.50% (HR = 2.97, 95% CI: 1.77–4.99, *P* < 0.001) and GWE < 86.99% (HR = 4.96, 95% CI: 2.93–8.40, *P* < 0.001) remained independently associated with mortality. Notably, NT-proBNP was not significant in the presence of LASr and GWE (*P* = 0.185), suggesting that these functional parameters capture aspects of disease severity beyond natriuretic peptide levels. MRV was marginally significant (HR = 1.015 per 1 mL, 95% CI: 1.001–1.030, *P* = 0.037), consistent with its known prognostic role in SMR.

### Sensitivity analysis: exclusion of mild MR

To determine whether the inclusion of mild MR influenced our findings, we repeated the main analyses after excluding patients with MRV < 30 mL, leaving a cohort of 203 patients with moderate-to-severe SMR (67 events, 33.0%). The optimal cutoffs for LASr and GWE in this restricted cohort, derived from ROC analysis, were 9.5% and 86.92%, respectively (Supplementary Figure S2). Kaplan–Meier curves demonstrated that both LASr < 9.5% (Supplementary Figure S3A) and GWE < 86.92% (Supplementary Figure S3B) remained associated with mortality, with log-rank *P* < 0.001 for both (LASr: HR = 4.283, 95% CI: 2.639–6.950; GWE: HR = 5.022, 95% CI: 2.859–8.820).

Univariate Cox regression analyses in this restricted cohort (Supplementary Table S5) showed that LASr < 9.5% (HR = 4.283, 95% CI: 2.639–6.950, *P* < 0.001) and GWE < 86.92% (HR = 5.022, 95% CI: 2.859–8.820, *P* < 0.001) were associated with mortality.

In multivariable Cox regression models restricted to the moderate-to-severe SMR cohort (Table 3), LASr < 9.5% (HR = 3.63, 95% CI: 2.10–6.30, *P* < 0.001) and GWE < 86.92% (HR = 3.44, 95% CI: 1.91–6.20, *P* < 0.001) remained independently associated with mortality after adjustment for LVEF, AF, and LAVI (Model 3). The addition of NYHA class (Model 4) did not materially alter these estimates (LASr: HR = 3.51, 95% CI: 2.02–6.12; GWE: HR = 3.38, 95% CI: 1.87–6.10). In a fully adjusted model (Supplementary Table S6) additionally including age, sex, and LVMI, LASr and GWE remained significant (LASr: HR = 3.63, 95% CI: 2.08–6.35; GWE: HR = 3.41, 95% CI: 1.87–6.20, both *P* < 0.001).

**Table 3:** Multivariable Cox regression models for predicting all-cause mortality in patients with moderate-to-severe SMR (MRV ≥ 30 mL).

Variable	Model 0 (Baseline)	Model 1 (+LASr)	Model 2 (+GWE)	Model 3 (+LASr + GWE)	Model 4 (+LASr + GWE + NYHA)
LVEF < 50%	2.749 (1.253–6.034)*	2.864 (1.304–6.288)**	1.921 (0.866–4.260)	2.130 (0.957–4.743)	1.978 (0.870–4.497)
Atrial fibrillation	0.811 (0.472–1.393)	0.931 (0.539–1.606)	0.875 (0.507–1.511)	0.832 (0.479–1.445)	0.823 (0.474–1.428)
LAVI > 48 mL/m^2^	1.211 (0.717–2.047)	0.669 (0.372–1.204)	1.096 (0.646–1.861)	0.671 (0.371–1.215)	0.673 (0.373–1.213)
LASr < 9.5%	—	4.990 (2.917–8.536)***	—	3.634 (2.096–6.299) ***	3.513 (2.017–6.118) ***
GWE < 86.92%	—	—	4.510 (2.545–7.993) ***	3.438 (1.907–6.199) ***	3.379 (1.871–6.100) ***
NYHA III–IV	—	—	—	—	1.249 (0.708–2.205)

Data are HR (95% CI). Model 0 (Baseline): adjusted for LVEF, AF, and LAVI. Model 1: Model 0 + LASr. Model 2: Model 0 + GWE. Model 3: Model 0 + LASr+GWE. Model 4: Model 3 + NYHA.

*P < 0.05, **P < 0.01, ***P < 0.001.

LASr, left atrial reservoir strain; GWE, global work efficiency; LAVI, left atrial volume index; LVEF, left ventricular ejection fraction; AF, atrial fibrillation; NYHA, New York Heart Association functional classification.

Mediation analysis in the moderate-to-severe SMR cohort showed findings consistent with those in the full cohort, with an indirect effect of GWE of −0.210 (95% CI: −0.371 to −0.110) and a mediation proportion of 18.0% (95% CI: 8.5%–32.0%) (Supplementary Figure S4).

### Subgroup analysis

**Figure 4:**
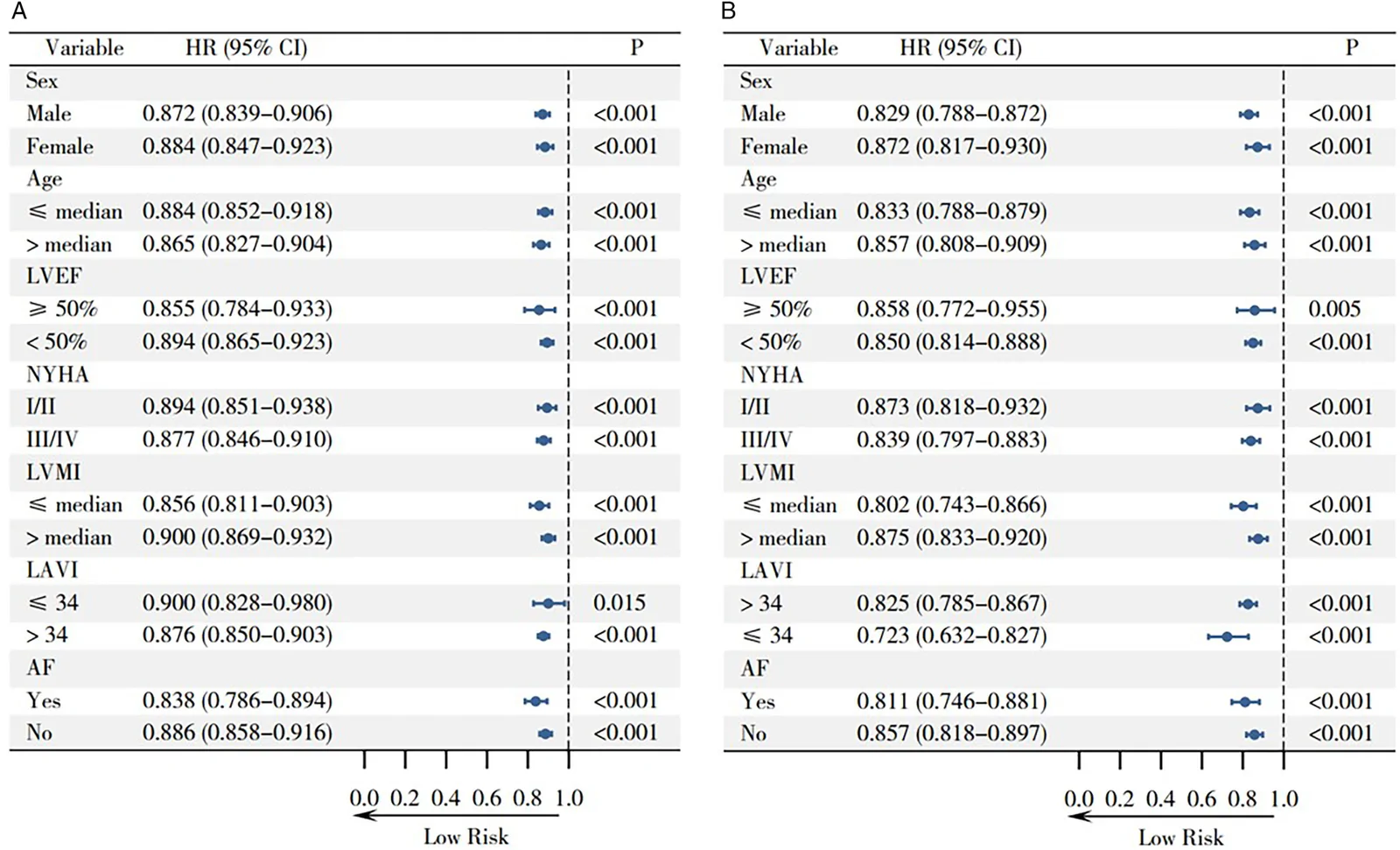
Subgroup analysis of LASr **(A)** and GWE **(B)** for the risk of all-cause mortality. Forest plots showing hazard ratios (HRs) and 95% confidence intervals (CIs) for the association of LASr **(A)** and GWE **(B)** with all-cause mortality across various clinical subgroups. The subgroups were stratified by sex (male vs. female), age (≤ 70 years vs. > 70 years), LVEF (≥ 50% vs. < 50%), NYHA functional class (I/II vs. III/IV), LVMI (≤ 144.55 g/m^2^ vs. > 144.55 g/m^2^), LAVI (≤ 34 mL/m^2^ vs. > 34 mL/m^2^), and AF status (yes vs. no). HRs were derived from univariate Cox regression models within each subgroup and are presented without multivariable adjustment. The vertical dashed line represents the null effect (HR = 1.0). Horizontal lines indicate 95% CIs, and the point estimates are represented by squares. *P* values for interaction are shown on the right side of the forest plot (LASr: range 0.065–0.930; GWE: range 0.107–0.926; all *P* for interaction > 0.05), indicating no significant effect modification across subgroups. Interaction tests were performed by including the product term between each stratification variable and LASr or GWE in separate Cox models. LASr, left atrial reservoir strain; GWE, global work efficiency; HR, hazard ratio; CI, confidence interval; LVEF, left ventricular ejection fraction; NYHA, New York Heart Association; LVMI, left ventricular mass index; LAVI, left atrial volume index; AF, atrial fibrillation.

Figure 4 shows forest plots of subgroup analyses. HRs were derived from univariate Cox regression models within each subgroup and are presented without multivariable adjustment. The prognostic effects of LASr and GWE were consistent across subgroups defined by sex, age, LVEF, NYHA class, LVMI, LAVI, and AF status. For LASr, HRs ranged from 0.723 (95% CI: 0.632–0.827) in patients with LAVI ≤34 to 0.875 (95% CI: 0.833–0.920) in patients with LVMI > median. For GWE, HRs ranged from 0.838 (95% CI: 0.786–0.894) in patients with AF to 0.900 (95% CI: 0.869–0.932) in patients with LVMI > median. Supplementary Table S7 lists interaction *P*-values, all >0.05 (range: 0.065–0.930), indicating no significant effect modification.

### Subgroup analysis of EROA

Subgroup analyses for EROA showed that it remained significantly associated with mortality across most strata (Supplementary Table S8), except in the LVEF ≥ 50% subgroup (HR = 1.041, 95% CI: 0.993–1.092, *P* = 0.097). Interaction *P* values were all > 0.05 except for LAVI at the 48 mL/m^2^ cutoff (*P* = 0.021). Severe LA dilation (LAVI > 48 mL/m^2^) is a strong prognostic marker in heart failure and may attenuate the incremental value of EROA, suggesting that LA remodeling dominates the prognostic signal in advanced disease stages.

### Mediation analysis

**Figure 5:**
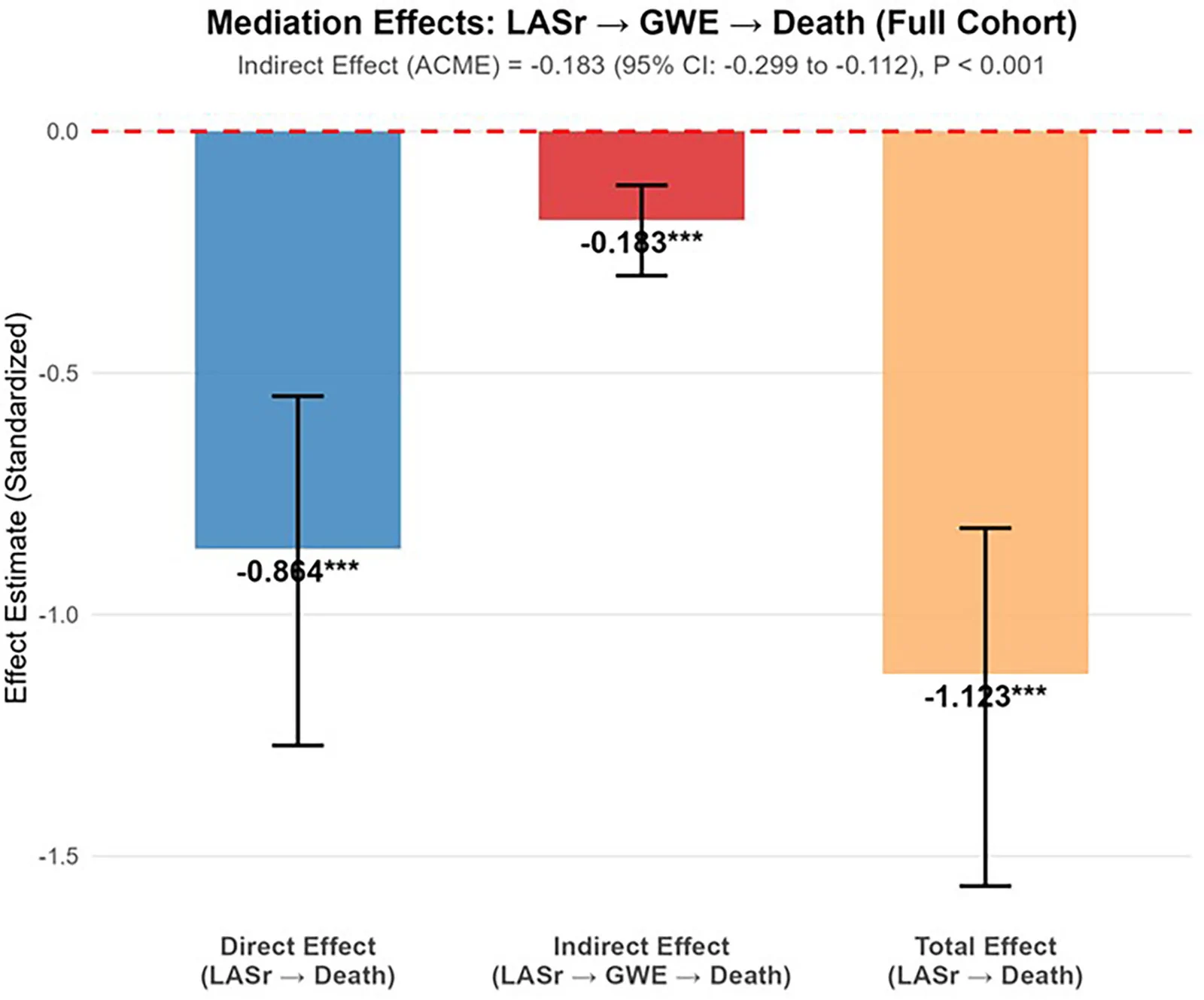
Exploratory mediation analysis of LASr on all-cause mortality through GWE in the full cohort. Bar plot showing the decomposition of the total association of LASr with all-cause mortality into direct and indirect components, as estimated from a statistical mediation model. All effect estimates are standardized; negative values indicate that higher LASr is associated with lower mortality risk (protective effect). The Total Effect (−1.123, 95% CI: −1.567 to −0.833) represents the overall association between LASr and mortality. The Direct Effect (ADE) (−0.864, 95% CI: −1.287 to −0.523) represents the portion of the LASr–mortality association not explained by GWE. The Indirect Effect (ACME) (−0.183, 95% CI: −0.305 to −0.112, *P* < 0.001) represents the portion of the association statistically accounted for by GWE. The Proportion Mediated was 16.3% (95% CI: 8.5% to 28.3%). All estimates are adjusted for age, sex, NYHA class, LVMI, LAVI, MRV, and LVEF. Error bars indicate 95% confidence intervals derived from 1,000 bootstrap resamples. The dashed red line at zero indicates no effect. This mediation analysis is exploratory and hypothesis-generating. LASr and GWE were measured at the same time point, and causal interpretations should be avoided. The findings are presented as a statistical framework to generate hypotheses for future prospective validation. ADE, average direct effect; ACME, average causal mediation effect; CI, confidence interval; GWE, global work efficiency; LASr, left atrial reservoir strain.

Figure 5 presents the exploratory mediation analysis examining the role of GWE in the LASr–mortality association. The total association of LASr with mortality was −1.123 (95% CI: −1.567 to −0.833). The direct effect (ADE) was −0.864 (95% CI: −1.287 to −0.523). The indirect effect (ACME) was −0.183 (95% CI: −0.305 to −0.112, *P* < 0.001). The proportion of the total effect mediated by GWE was 16.3% (95% CI: 8.5% to 28.3%). These findings are consistent with an exploratory mediation framework in which GWE partially explains the LASr–mortality association.

To assess the robustness of the mediation findings across the SMR severity spectrum, we repeated the exploratory mediation analysis in the moderate-to-severe SMR cohort (MRV ≥ 30 mL, *n* = 203, events=67). The indirect effect through GWE remained significant (ACME=−0.210, 95% CI: −0.371 to −0.110, *P* = 0.001), with a mediation proportion of 18.0% (95% CI: 8.5%–32.0%), consistent with the full-cohort estimate of 16.3%. The modest decrease in the proportion statistically accounted for by GWE was attributable to an increased direct association of LASr in the restricted cohort, rather than a true attenuation of the indirect pathway. These findings collectively suggest that the mediation framework is robust and not materially influenced by the inclusion or exclusion of patients with mild MR.

## Discussion

This study is the first to systematically investigate the interplay between LASr and GWE, and to explore, within a statistical mediation framework, whether GWE partially explains the LASr–mortality association, in medically managed SMR patients without valve intervention. While prior studies have established the individual prognostic value of LASr and GWE in SMR, the combined assessment of these two parameters and the potential statistical framework associated with left atrial dysfunction to impaired myocardial work efficiency have not been explored. In this cohort of 399 medically treated SMR patients, we demonstrated that both LASr and GWE independently predicted all-cause mortality, and that GWE partially statistically accounted for this association, accounting for 16.3% (95% CI: 8.5% to 28.3%) of the total effect.

The prognostic value of myocardial work parameters in SMR has been previously established. In a cohort of 373 SMR patients with a median LVEF of 34% (IQR: 25%–49%), Yedidya et al. demonstrated that reduced LV GWI, GCW, and GWW were independently associated with excess mortality (10). Notably, patients with severe SMR exhibited paradoxically higher GWE (82% vs. 76%, *P* = 0.001) compared with those with mild SMR, reflecting LV unloading in the low-pressure left atrial chamber. This paradoxical elevation, however, does not imply preserved myocardial function, as these patients still had worse long-term survival. In our cohort, non-survivors had significantly lower GWE than survivors—a finding that likely reflects more advanced intrinsic myocardial impairment, consistent with their lower LVEF, higher LVMI, and lower LASr, rather than a failure of unloading *per se*. Collectively, these observations are consistent with the view that while unloading may be associated with higher GWE in severe SMR, GWE is independently associated with prognosis across a broader SMR population. In the general population, GWE has also been shown to be associated with cardiovascular events, further supporting its prognostic relevance beyond selected cohorts (13). Our findings extend prior evidence by confirming that GWE provides independent prognostic information beyond LVEF and conventional parameters across a wider LVEF spectrum (20%–70%), with significant improvement in model fit upon its addition (AIC reduction from 1,137.37 to 1,068.10). Importantly, the prognostic value of LASr and GWE was not attenuated when the analysis was restricted to patients with moderate-to-severe SMR (MRV ≥ 30 mL). In this subgroup, LASr and GWE remained independently associated with mortality, with effect sizes comparable to those observed in the full cohort, suggesting that our findings are not merely driven by the inclusion of mild MR cases. The robustness of our findings was further supported by an extended multivariable model that adjusted for NT-proBNP, eGFR, SBP, ischemic etiology, TAPSE, TR velocity, and GDMT score. Even after this comprehensive adjustment, LASr and GWE remained associated with mortality, whereas NT-proBNP was no longer significant, suggesting that LASr and GWE provide prognostic information beyond conventional biomarkers of heart failure severity. In supplementary analyses, GWE demonstrated incremental prognostic value beyond GLS, with improved model fit and discrimination. The GWE component independent of GLS remained significantly associated with mortality (HR = 0.89, *P* < 0.001).

The independent prognostic value of LASr in SMR has been similarly established. Stassen et al. reported that LASr ≥9.8% was independently associated with lower all-cause mortality (HR 0.499, 95% CI 0.386–0.645; *P* < 0.001) after multivariable adjustment (18). This finding has been further validated in the atrial functional MR (AFMR) subtype, a distinct SMR phenotype characterized by preserved LVEF, where LASr was shown to impact clinical outcomes (17). In a study of 515 AFMR patients, low LASr was associated with a 2.3-fold higher risk of death (HR 2.3, 95% CI 1.6–3.5) after multivariable adjustment, and importantly, this association was independent of LA volume—suggesting that LA function, rather than size, drives prognosis in AFMR (17). Furthermore, Carvalheiro et al. confirmed that LASr independently predicted all-cause mortality in functional MR (adjusted HR 0.887, *P* = 0.039), with further risk stratification achieved by combining LASr with tricuspid regurgitation velocity (24). Our results are consistent: impaired LASr was associated with a higher mortality risk (HR 5.404 for LASr <13.50% vs. ≥13.50% in univariate analysis; HR 0.891 per 1% increase in multivariable Model 4). Notably, the prognostic value of LA strain extends to acute heart failure, where Barki et al. demonstrated that LA strain dynamics predict rehospitalization across the LVEF spectrum, supporting its broader utility as a marker of LA dysfunction (25). Together, these findings support integrating both GWE and LASr into routine echocardiographic assessment for refining risk stratification in SMR patients. Our IDI findings showed that adding LASr to GWE did not improve risk reclassification (IDI = −0.027, *P* = 0.016), consistent with their moderate correlation (*r* = 0.595), suggesting that GWE captures most of the prognostic information from LASr in this cohort. Having established their independent prognostic value, we next explored whether these two parameters are statistically associated within our analytical framework. Specifically, we examined whether GWE statistically accounts for part of the relationship between LASr and mortality. AF may influence LASr measurements, but our subgroup analysis showed consistent prognostic value in both AF and non-AF patients (*P* for interaction=0.809).

Among the three SMR phenotypes, dual functional MR was not only the most prevalent but also carried the worst prognosis, with a mortality rate of 47.4% (81/171) compared with 10.9% (7/64) in AFMR and 19.7% (15/76) in VFMR. Patients with dual FMR also had the most severely impaired LASr and GWE. This observation is consistent with recent registry data indicating that dual FMR is associated with the highest risk of all-cause mortality and heart failure hospitalization among SMR phenotypes (26). The dual phenotype represents a mixed form of functional MR in which atrial-driven mechanisms (left atrial enlargement and annular dilatation) coexist with ventricular-driven mechanisms (left ventricular remodeling and leaflet tethering), and their combination may impose a synergistic hemodynamic burden that exceeds the sum of its individual components. These findings have important clinical implications: SMR patients with mixed phenotypes may require more aggressive monitoring and earlier consideration for advanced therapies.

A key novel finding of our study is the statistical mediation observed, with an indirect effect of −0.183 (95% CI: −0.305 to −0.112) and a mediation proportion of 16.3% (95% CI: 8.5% to 28.3%). Interpreted within the constraints of the cross-sectional design, this finding is consistent with the hypothesis that left atrial dysfunction (as assessed by reduced LASr) may be statistically related to impaired myocardial work efficiency (lower GWE) and is associated with adverse outcomes. However, as LASr and GWE were measured at the same time point, we cannot establish a temporal or causal sequence from this analysis alone. Left atrial (LA) dysfunction, as reflected by reduced LASr, may impair LV filling and elevate LA pressure, which in turn compromises myocardial work efficiency (lower GWE) and ultimately leads to adverse outcomes (18, 27). LA reservoir function is a key determinant of LV filling, and its impairment directly affects transmitral flow dynamics and LV diastolic performance (27, 28). In SMR, chronic volume overload from regurgitation leads to LA remodeling and dysfunction, which may further exacerbate the hemodynamic burden on the LV (17, 18). The progressive decline in LA function across heart failure stages has been shown to correlate with worsening LV filling and increased filling pressure (27, 29). Given that SMR imposes a volume load on the LV—where the regurgitant fraction contributes to total stroke volume without contributing to forward output—LVEF becomes a load-dependent parameter that may overestimate true contractile function (10). GWE, which integrates myocardial deformation with afterload, offers a more comprehensive assessment of LV performance independent of loading conditions (10, 30). In SMR, the unloading effect in the low-pressure left atrial chamber may paradoxically elevate GWE, yet impaired GWE still reflects underlying LV dysfunction and predicts worse outcomes (10). The partial mediation we observed suggests that while GWE explains a portion of the LASr–mortality association, LASr is also independently associated with mortality, potentially through other factors such as LA–LV interaction, neurohormonal activation, or pulmonary vascular consequences (18, 27). These findings highlight the interconnectedness of LA and LV function in SMR and support the integration of both LASr and GWE into comprehensive risk stratification. The recognition of this statistical mediation framework carries several potential clinical implications. However, the observed mediation proportion of 16.3% indicates partial mediation, suggesting that other factors—such as neurohormonal activation, atrial fibrosis, or pulmonary vascular remodeling—may also contribute to the remaining association between LASr and mortality (27, 31, 32). These interpretations are speculative and require prospective validation.

Our findings have several potential clinical implications. First, both LASr and GWE provide independent prognostic information beyond conventional parameters, supporting their integration into routine echocardiographic assessment in SMR patients. Specifically, our findings suggest that a LASr <13.50% or GWE <86.99% may help identify patients at higher risk of mortality. However, these cutoffs were derived from our single-center cohort and require external validation in independent populations before they can be applied to clinical decision-making. Second, the mediation framework generates the hypothesis that changes in GWE—whether through guideline-directed medical therapy or mitral valve intervention—may be associated with improved outcomes in patients with impaired LASr, although this hypothesis requires prospective validation. In line with this, data from the CRT population have shown that improvement in MR severity is independently associated with an increase in LASr, and that an increase in LASr is associated with lower mortality (33). Furthermore, in patients with SMR undergoing TEER, baseline GWE was independently associated with improvement in forward stroke volume index (≥20%) after the procedure, independent of LV systolic function (34). The proposed cutoff values (LASr <13.50%, GWE <86.99%) were derived from our single-center cohort and require external validation in independent populations before they can be applied to clinical decision-making.

The subgroup analyses of EROA further support the robustness of MR severity as a prognostic marker in SMR. EROA remained significantly associated with mortality across most clinical and echocardiographic strata, with no significant interactions detected for sex, age, NYHA class, AF, or LVMI. Notably, the interaction with LAVI at the 48 mL/m^2^ cutoff (*P* = 0.021) suggests that severe left atrial dilation may attenuate the incremental prognostic value of EROA, possibly because LAVI > 48 mL/m^2^ itself represents an advanced disease state where additional information from EROA becomes relatively limited.

Several limitations should be acknowledged. First, this was a single-center retrospective study, which may introduce selection bias, and the sample size may limit the power of subgroup evaluations. Second, residual confounding cannot be excluded despite multivariable adjustment; the extended model had an EPV of 8.55 and should be interpreted as exploratory. Third, the mediation analysis was exploratory and hypothesis-generating; LASr and GWE were measured simultaneously, precluding causal inference, and the 16.3% mediation proportion suggests other unmeasured pathways (e.g., neurohormonal activation, atrial fibrosis, pulmonary vascular remodeling) likely contribute to the remaining effect. Fourth, the optimal cutoffs for LASr and GWE require external validation before clinical application. Fifth, GWE and LASr measurements require dedicated speckle-tracking software, and SMR phenotypes classification was based on echocardiographic criteria rather than invasive assessment, which may limit generalizability. Sixth, cause-specific death data were not available, and serial LASr measurements during follow-up were not systematically obtained, precluding analysis of temporal changes in LA function.

## Conclusions

In patients with medically managed SMR, both LASr and GWE are independently associated with all-cause mortality, and GWE partially statistically accounts for a portion of the LASr–mortality association in an exploratory mediation framework. These findings are consistent with the hypothesis that left atrial dysfunction and reduced myocardial work efficiency are related aspects of the same pathophysiological process. Assessment of both parameters may refine risk stratification in SMR patients, although the proposed cutoffs require external validation before they can be recommended for routine clinical use.

## Data Availability

The datasets generated and/or analyzed during the current study are not publicly available due to limitations of ethical approval involving patient data and anonymity, but are available from the corresponding author on reasonable request. Requests to access these datasets should be directed to cylkl0121@163.com.
