# Supplementary material for: Prognostic value of left atrial reservoir strain and global work efficiency in medically managed secondary mitral regurgitation: a mediation analysis
Source: Front Cardiovasc Med. 2026 Sep 3;13:1924498. doi: 10.3389/fcvm.2026.1924498 (PMC13581840; doi:10.3389/fcvm.2026.1924498)
Supplement: Supplementary file 1 [file Datasheet1.docx]

Supplementary Material

**Supplementary Figure S1. Receiver operating characteristic (ROC) curves of LASr and GWE for predicting all-cause mortality.** ROC curves comparing the discriminative ability of left atrial reservoir strain (LASr, blue line) and global work efficiency (GWE, red line). LASr showed an AUC of 0.779 (95% CI: 0.728–0.830), while GWE showed an AUC of 0.788 (95% CI: 0.739–0.838). The DeLong test for paired ROC curves indicated no significant difference between the two AUCs (P = 0.7594). The gray dashed diagonal line represents the reference line (AUC = 0.5). Optimal cut-off values for predicting mortality were 13.50% for LASr (sensitivity: 0.689, specificity: 0.720) and 86.99% for GWE (sensitivity: 0.757, specificity: 0.693).

**Supplementary Figure S2. Receiver operating characteristic (ROC) curves of LASr and GWE for predicting all-cause mortality in patients with moderate-to-severe secondary mitral regurgitation (MRV ≥ 30 mL).**

The area under the curve (AUC) for LASr was 0.744 (95% CI: 0.671–0.816), with an optimal cutoff of 9.5% (sensitivity: 0.522, specificity: 0.846). The AUC for GWE was 0.800 (95% CI: 0.730–0.869), with an optimal cutoff of 86.92% (sensitivity: 0.761, specificity: 0.706). Both parameters demonstrated good discriminative ability for predicting mortality in this cohort. The DeLong test showed no statistically significant difference between the two AUCs (P = 0.174), indicating comparable prognostic performance of LASr and GWE.

LASr, left atrial reservoir strain; GWE, global work efficiency; MRV, mitral regurgitation volume; CI, confidence interval; AUC, area under the curve.

**Supplementary Figure S3. Kaplan-Meier survival curves for all-cause mortality according to LASr and GWE levels in patients with moderate-to-severe secondary mitral regurgitation (MRV ≥ 30 mL).**

(A) Survival curves for patients stratified by LASr levels (high: ≥9.5%; low: <9.5%). (B) Survival curves for patients stratified by GWE levels (high: ≥86.92%; low: <86.92%). Log-rank tests demonstrated significantly lower survival probabilities in the low LASr and low GWE groups compared with the high LASr and high GWE groups, respectively (both P < 0.001). Hazard ratios (HRs) were calculated using univariate Cox regression analysis. LASr low vs. high: HR = 4.283 (95% CI: 2.639–6.950), P < 0.001. GWE low vs. high: HR = 5.022 (95% CI: 2.859–8.820), P < 0.001. Number at risk at each time point is shown below the curves. MRV, mitral regurgitation volume; LASr, left atrial reservoir strain; GWE, global work efficiency.

**Supplementary Figure S4. Exploratory mediation analysis of the LASr–mortality association through GWE in patients with moderate‑to‑severe secondary mitral regurgitation (MRV ≥ 30 mL).**

Bar plot showing the decomposition of the total association of LASr with all-cause mortality into direct and indirect components, as estimated from a statistical mediation model, adjusted for LVEF < 50%, AF, LAVI > 48 mL/m², and NYHA III–IV (n = 203, events = 67). All effect estimates are standardized; negative values indicate that higher LASr is associated with lower mortality risk (protective effect). The Total Effect (c) was −1.164 (95% CI: −1.799 to −0.831, P < 0.001), representing the overall association between LASr and mortality. The Direct Effect (ADE, c′) was −0.872 (95% CI: −1.577 to −0.505, P = 0.001), representing the portion of the LASr–mortality association not explained by GWE. The Indirect Effect (ACME, a × b) through GWE was −0.210 (95% CI: −0.371 to −0.110, P = 0.001), representing the portion of the association statistically accounted for by GWE. The Proportion Mediated was 18.0% (95% CI: 8.5% to 32.0%, P = 0.005). Error bars indicate 95% confidence intervals derived from 1000 bootstrap resamples. The dashed red line at zero indicates no effect. In residual‑based validation, the GWE residual remained significantly associated with mortality (HR = 0.621 per 1‑SD increase, 95% CI: 0.488–0.791, P < 0.001), corroborating the mediation findings.

Note: This mediation analysis is exploratory and hypothesis‑generating. LASr and GWE were measured at the same time point, and causal interpretations should be avoided. The findings are presented as a statistical framework to generate hypotheses for future prospective validation.

Abbreviations: ADE, Average Direct Effect; ACME, Average Causal Mediation Effect; CI, Confidence Interval; GWE, Global Work Efficiency; LASr, Left Atrial Reservoir Strain; LVEF, Left Ventricular Ejection Fraction; AF, Atrial Fibrillation; LAVI, Left Atrial Volume Index; NYHA, New York Heart Association functional classification; HR, hazard ratio; SD, standard deviation.
