# Supplementary material for: Prognostic value of left atrial reservoir strain and global work efficiency in medically managed secondary mitral regurgitation: a mediation analysis
Source: Front Cardiovasc Med. 2026 Sep 3;13:1924498. doi: 10.3389/fcvm.2026.1924498 (PMC13581840; doi:10.3389/fcvm.2026.1924498)
Supplement: Supplementary file 2 [file Table1.docx]

Supplementary Material

**Supplementary Table** **S**1**.** ROC analysis for predicting all-cause mortality

| Variable | AUC | 95% CI | P value |
| --- | --- | --- | --- |
| GWE | 0.788 | 0.739–0.838 | <0.001 |
| LASr | 0.779 | 0.728–0.830 | <0.001 |
| GWW | 0.704 | 0.646–0.762 | <0.001 |
| GLS | 0.688 | 0.632-0.744 | <0.001 |
| LVEF | 0.676 | 0.617–0.735 | <0.001 |
| MRV | 0.675 | 0.619–0.731 | <0.001 |
| GWI | 0.643 | 0.585–0.701 | <0.001 |
| GCW | 0.616 | 0.557–0.675 | <0.001 |

Abbreviations: AUC, area under the curve; CI, confidence interval; GWE, global work efficiency; LASr, left atrial reservoir strain; GWW, global wasted work; GLS, Left Ventricular Global Longitudinal Strain; LVEF, left ventricular ejection fraction; MRV, mitral regurgitation volume; GWI, global work index; GCW, global constructive work.

**Supplementary Table** **S**2**. Model fit comparisons for multivariable Cox regression models**

| Model | AIC | C‑index | −2 Log L | LR χ² | Δχ² (vs. previous) | df | P for Δχ² |
| --- | --- | --- | --- | --- | --- | --- | --- |
| Model 1 (Basic) | 1151.92 | 0.7033 | 1141.05 | 46.62 | — | 6 | — |
| Model 2 (+LVEF) | 1137.37 | 0.7172 | 1124.63 | 63.04 | 16.42 | 7 | < 0.001 |
| Model 3 (+GWE) | 1092.63 | 0.7809 | 1078.51 | 109.16 | 46.12 | 8 | < 0.001 |
| Model 4 (+LASr) | 1068.09 | 0.7988 | 1052.46 | 135.21 | 26.05 | 9 | < 0.001 |
| Model 5 (+Interaction) | 1070.00 | 0.7985 | 1052.40 | 135.27 | 0.06 | 10 | 0.764 |

Model 1: adjusted for sex, age, NYHA (III–IV vs. I–II), LVMI, LAVI, and MRV. Model 2: Model 1 + LVEF. Model 3: Model 2 + GWE. Model 4: Model 3 + LASr. Model 5: Model 4 + GWE×LASr interaction term.

Lower AIC and −2 Log Likelihood values indicate better model fit. Likelihood ratio χ² (LR χ²) and P values are calculated relative to the null model. Δχ² and P for Δχ² represent the improvement from the previous model. Bold indicates the best-fitting model. C-index values are Harrell’s concordance index; higher values indicate better discriminative ability.

Abbreviations: AIC, Akaike information criterion; C-index, Harrell’s concordance index; LR, likelihood ratio; df, degrees of freedom; LVEF, left ventricular ejection fraction; GWE, global work efficiency; LASr, left atrial reservoir strain; LVMI, left ventricular mass index; LAVI, left atrial volume index; MRV, mitral regurgitation volume; NYHA, New York Heart Association functional classification.

**Supplementary Table S3. Integrated Discrimination Improvement (IDI) for nested Cox models**

| Reference Model | Comparison Model | IDI (95% CI) | P value |
| --- | --- | --- | --- |
| Model 1 (Basic) | Model 3 (+GWE) | 0.061 (0.024–0.098) | < 0.001 |
| Model 1 (Basic) | Model 4 (+LASr) | 0.035 (−0.003–0.070) | 0.064 |
| Model 1 (Basic) | Model 5 (+Interaction) | 0.025 (−0.012–0.060) | 0.177 |
| Model 2 (+LVEF) | Model 3 (+GWE) | 0.055 (0.023–0.088) | 0.001 |
| Model 2 (+LVEF) | Model 4 (+LASr) | 0.028 (−0.006–0.061) | 0.093 |
| Model 2 (+LVEF) | Model 5 (+Interaction) | 0.018 (−0.016–0.051) | 0.271 |
| Model 1 (Basic) | Model 2 (+LVEF) | 0.007 (−0.009–0.022) | 0.406 |
| Model 3 (+GWE) | Model 4 (+LASr) | −0.027 (−0.048–−0.006) | 0.016 |
| Model 3 (+GWE) | Model 5 (+Interaction) | −0.037 (−0.059–−0.015) | 0.001 |
| Model 4 (+LASr) | Model 5 (+Interaction) | −0.010 (−0.012–−0.008) | < 0.001 |

Model 1 (Basic): adjusted for sex, age, NYHA (III–IV vs. I–II), LVMI, LAVI, and MRV.
Model 2 (+LVEF): Model 1 + LVEF.
Model 3 (+GWE): Model 2 + GWE.
Model 4 (+LASr): Model 3 + LASr.
Model 5 (+Interaction): Model 4 + GWE×LASr interaction term.

IDI estimates and 95% confidence intervals were derived from 1000 bootstrap resamples. Positive IDI values indicate improved reclassification and discrimination of the comparison model over the reference model. Bold P values indicate statistically significant improvements at the P < 0.05 level. Negative IDI values indicate that the reference model outperforms the comparison model.

Abbreviations: IDI, integrated discrimination improvement; CI, confidence interval; GWE, global work efficiency; LASr, left atrial reservoir strain; LVEF, left ventricular ejection fraction.

**Supplementary Table** **S4. Multivariable Cox regression analysis for all-cause mortality in the full cohort with extended adjustment for clinical, biochemical, and echocardiographic covariates.**

| Variable | HR (95% CI) | P value |
| --- | --- | --- |
| LASr < 13.50% (high risk) | 2.972 (1.770–4.990) | < 0.001 |
| GWE < 86.99% (high risk) | 4.961 (2.932–8.395) | < 0.001 |
| log(NT‑proBNP) | 1.127 (0.944–1.346) | 0.185 |
| eGFR (per 1 mL/min/1.73m²) | 1.002 (0.993–1.011) | 0.724 |
| SBP (per 1 mmHg) | 0.988 (0.975–1.001) | 0.066 |
| Ischemic etiology | 1.360 (0.849–2.180) | 0.201 |
| Atrial fibrillation | 1.020 (0.647–1.609) | 0.931 |
| TAPSE (per 1 mm) | 1.049 (0.969–1.135) | 0.242 |
| TR velocity (per 1 m/s) | 0.744 (0.509–1.089) | 0.129 |
| GDMT score (per 1 point) | 0.785 (0.492–1.252) | 0.309 |
| MRV (per 1 mL) | 1.015 (1.001–1.030) | 0.037 |

Model adjusted for LASr, GWE, log (NT‑proBNP), eGFR, SBP, ischemic etiology, atrial fibrillation, TAPSE, TR velocity, GDMT score, and MRV. n = 364, events = 94, EPV = 8.55. Data are HR (95% CI).

Note: GDMT score was calculated by summing the use of ACEi/ARB/ARNI (1 point) and beta-blockers (1 point), yielding a score ranging from 0 to 2. This simplified scoring approach is based on the foundational framework of validated GDMT scoring systems, which identify RAAS inhibitors (ACEi/ARB/ARNI) and beta-blockers as the core mandatory components of guideline-directed medical therapy in heart failure. MRAs and SGLT2 inhibitors were not included in the score due to their extremely low prescription rates (<10% for MRAs; SGLT2 inhibitors had not been widely adopted during the study enrollment period)

LASr, left atrial reservoir strain; GWE, global work efficiency; NT‑proBNP, N‑terminal pro‑B‑type natriuretic peptide; eGFR, estimated glomerular filtration rate; SBP, systolic blood pressure; TAPSE, tricuspid annular plane systolic excursion; TR, tricuspid regurgitation; GDMT, guideline‑directed medical therapy; MRV, mitral regurgitation volume; HR, hazard ratio; CI, confidence interval; EPV, events per variable.

**Supplementary Table** **S5. Univariate Cox regression analysis for all-cause mortality in patients with moderate-to-severe secondary mitral regurgitation (MRV ≥ 30 mL)**

| Variable | HR (95% CI) | P value | |  |
| --- | --- | --- | --- | --- |
| **Demographic and clinical characteristics** | | | |  |
| Age (per 1-year increase) | 1.010 (0.990–1.029) | 0.331 | |  |
| Sex (male vs. female) | 0.884 (0.530–1.473) | 0.636 | |  |
| NYHA class III–IV (vs. I–II) | 1.972 (1.149–3.385) | 0.014* | |  |
| Atrial fibrillation | 0.820 (0.478–1.409) | 0.473 | | |
| **Echocardiographic parameters** | | | | |
| LVEF < 50% | 2.771 (1.263–6.078) | 0.011* | | |
| LVMI (per 1 g/m² increase) | 1.006 (1.000–1.011) | 0.043* | | |
| LAVI > 48 mL/m² | 1.253 (0.741–2.117) | 0.4 | | |
| MRV (per 1 mL increase) | 1.026 (1.008–1.045) | 0.004** | | |
| **Myocardial work and strain parameters** | | | | |
| LASr < 9.5% | 4.283 (2.639–6.950) | < 0.001*** | | |
| GWE < 86.92% | 5.022 (2.859–8.820) | < 0.001*** |  |  |

Data are HR (95% CI) from univariate Cox models. LASr and GWE were dichotomized at optimal cutoffs derived from Youden's index (9.5% and 86.92%, respectively). LVEF < 50%, LAVI > 48 mL/m², NYHA III–IV, and AF were coded as binary variables. *P < 0.05, **P < 0.01, ***P < 0.001. For abbreviations, see Table 1.

**Supplementary Table** **S6. Multivariable Cox regression analysis for all‑cause mortality with additional adjustment for age, sex, and LVMI in patients with moderate‑to‑severe secondary mitral regurgitation (MRV ≥ 30 mL).**

| Variable | HR (95% CI) | P value |
| --- | --- | --- |
| **Echocardiographic parameters** |  |  |
| LVEF < 50% | 2.148 (0.892–5.169) | 0.088 |
| Atrial fibrillation | 0.846 (0.480–1.494) | 0.565 |
| LAVI > 48 mL/m² | 0.670 (0.368–1.218) | 0.189 |
| **Myocardial work and strain parameters** |  |  |
| LASr < 9.5% (high risk) | 3.629 (2.075–6.347) | < 0.001* |
| GWE < 86.92% (high risk) | 3.408 (1.873–6.204) | < 0.001* |
| **Additional adjusted covariates** |  |  |
| Age (per 1 year) | 1.004 (0.983–1.025) | 0.737 |
| Male (vs. female) | 1.082 (0.632–1.853) | 0.774 |
| LVMI (per 1 g/m²) | 1.001 (0.995–1.007) | 0.865 |

Model adjusted for LVEF < 50%, AF, LAVI > 48 mL/m², LASr < 9.5%, GWE < 86.92%, age (per 1 year), sex (male vs. female), and LVMI (per 1 g/m²).
Data are presented as hazard ratio (HR) with 95% confidence interval (CI).
*P < 0.001.
MRV, mitral regurgitation volume; LVEF, left ventricular ejection fraction; LAVI, left atrial volume index; LASr, left atrial reservoir strain; GWE, global work efficiency; LVMI, left ventricular mass index; AF, atrial fibrillation

**Supplementary Table** **S**7. Interaction P-values for Subgroup Analyses

| Variable | LASr × Subgroup | GWE × Subgroup |
| --- | --- | --- |
| Sex | 0.5276 | 0.2008 |
| Age | 0.4907 | 0.4085 |
| LVEF | 0.3543 | 0.9295 |
| NYHA | 0.5518 | 0.3126 |
| LVMI | 0.1138 | 0.0669 |
| LAVI | 0.5754 | 0.0649 |
| AF | 0.0949 | 0.1565 |

Abbreviations: P-values < 0.05 indicate significant interaction (bold). Interaction tests showed no significant effect modification for LASr and GWE across all subgroups.

**Supplementary Table** S8 Subgroup analysis for the association of EROA with all cause mortality

| Subgroup | Level | N | Events | HR (95% CI) | P value |
| --- | --- | --- | --- | --- | --- |
| Overall | All patients | 399 | 103 | 1.032 (1.022–1.043) | < 0.001 |
| Sex | Male | 265 | 66 | 1.037 (1.023–1.051) | < 0.001 |
|  | Female | 134 | 37 | 1.026 (1.009–1.043) | 0.003 |
| Age | ≤ 70 years | 210 | 55 | 1.032 (1.016–1.048) | < 0.001 |
|  | > 70 years | 189 | 48 | 1.033 (1.019–1.047) | < 0.001 |
| LVEF | ≥ 50% | 117 | 11 | 1.041 (0.993–1.092) | 0.097 |
|  | < 50% | 282 | 92 | 1.025 (1.014–1.037) | < 0.001 |
| NYHA | I–II | 185 | 31 | 1.038 (1.020–1.055) | < 0.001 |
|  | III–IV | 214 | 72 | 1.025 (1.011–1.039) | < 0.001 |
| AF | No | 271 | 74 | 1.034 (1.021–1.047) | < 0.001 |
|  | Yes | 128 | 29 | 1.029 (1.009–1.050) | 0.005 |
| LVMI | ≤ 144.55 g/m² | 200 | 33 | 1.035 (1.015–1.054) | < 0.001 |
|  | > 144.55 g/m² | 199 | 70 | 1.025 (1.012–1.039) | < 0.001 |
| LAVI_1_ | ≤ 34 mL/m² | 78 | 15 | 1.065 (1.024–1.107) | 0.002 |
|  | > 34 mL/m² | 321 | 88 | 1.029 (1.018–1.041) | < 0.001 |
| LAVI_2_ | ≤ 48 mL/m² | 189 | 35 | 1.057 (1.031–1.084) | < 0.001 |
|  | > 48 mL/m² | 210 | 68 | 1.022 (1.008–1.036) | 0.002 |

**Abbreviations:** Data are presented as hazard ratio (HR) with 95% confidence interval (CI) per 1 mm² increase in EROA. HRs were derived from univariate Cox regression models within each subgroup. Interaction P values were calculated by including the product term between each stratification variable and EROA in separate Cox models: sex (P = 0.240), age (P = 0.988), LVEF (P = 0.484), NYHA (P = 0.312), AF (P = 0.809), LVMI (P = 0.428), LAVI_34 (P = 0.101), and LAVI_48 (P = 0.021). EROA, effective regurgitant orifice area; LVEF, left ventricular ejection fraction; NYHA, New York Heart Association functional classification; AF, atrial fibrillation; LVMI, left ventricular mass index; LAVI, left atrial volume index; CI, confidence interval.
